# Digital Technical and Informal Resources of Breast Cancer Patients From 2012 to 2020: Questionnaire-Based Longitudinal Trend Study

**DOI:** 10.2196/20964

**Published:** 2021-11-18

**Authors:** Christoph A Mallmann, Christian M Domröse, Lars Schröder, David Engelhardt, Frederik Bach, Helena Rueckel, Alina Abramian, Christina Kaiser, Alexander Mustea, Andree Faridi, Wolfram Malter, Peter Mallmann, Christian Rudlowski, Oliver Zivanovic, Michael R Mallmann

**Affiliations:** 1 Department of Surgery University Hospital of Cologne Cologne Germany; 2 Center of Integrated Oncology Aachen, Köln, Bonn, Düsseldorf Cologne Germany; 3 Department of Obstetrics & Gynecology University Hospital of Cologne Cologne Germany; 4 Department of Obstetrics & Gynecology Klinikum Hanau Hanau Germany; 5 Breast Center University Hospital of Bonn Bonn Germany; 6 Department of Gynecology & Gynecologic Oncology University Hospital of Bonn Bonn Germany; 7 Department of Obstetrics & Gynecology Evangelic Hospital Bergisch Gladbach Bergisch Gladbach Germany; 8 Department of Surgery Memorial Sloan-Kettering Cancer Center New York, NY United States

**Keywords:** digitalization, eHealth, breast cancer, internet

## Abstract

**Background:**

Digitalization offers enormous potential in medicine. In the era of digitalization, the development of the use of digital, technical, and informal resources of breast cancer patients and factors influencing the degree of digitization of patients has been insufficiently researched.

**Objective:**

The aim of this study was to assess the development of the use of digital technical and informal resources in a well-defined patient cohort.

**Methods:**

A longitudinal study on 513 breast cancer patients from 2012 to 2020 was conducted using a questionnaire that included the main aspects of the degree of digitalization, including digital device availability and use, stationary and mobile internet access and use, and communication and information seeking regarding breast cancer diagnosis and treatment.

**Results:**

The majority of patients (421/513, 82.1%) owned the technical resources to benefit from eHealth, used the internet to obtain information (292/509, 57.4%), and were willing to use new eHealth solutions (379/426, 89%). Two-thirds of the patients discussed information about their cancer on the internet with their doctor, one-third found additional treatment options on the internet, and 15.3% (44/287) of the patients stated that this had changed their cancer therapy. The degree of digitization is increasing yet still significantly depends on 3 factors: (1) age (whereas 100% [39/39] of the <59-year-old group used the internet in 2020, 92% of the 60 to 69-year-old group [11/12] and only 47% [6/13] of the >70-year-old group used the internet), (2) education (internet use significantly depended on education, as only 51.8% [59/114] of patients with primary school education used the internet, but 82.4% [126/153] with middle school education and 90.3% [213/236] with high school education used the internet; *P*<.001), and (3) household size (67.7% [111/164] of patients living alone used the internet, whereas 84.7% [287/339] of patients living in a house with ≥2 people used the internet; *P*<.001).

**Conclusions:**

To implement digital solutions in health care, knowledge of the composition and degree of the use of digital technical and informal resources of the patient group for which the respective solution is developed is crucial for success.

**Trial Registration:**

German Register of Clinical Studies DRKS00012364; https://www.drks.de/drks_web/navigate.do?navigationId=trial.HTML&TRIAL_ID=DRKS00012364

## Introduction

Catalyzed by the development of the internet, changes in digitalization are occurring more rapidly in both public and private life. Digitalization with its influence on information seeking, decision-making properties of patients, therapy monitoring, and patient-physician interaction will likely change the health sector in both developed and developing countries [1-3]. Concepts of digitalization such as digital patient diaries and digital side-effect management have become part of many clinical trials [4-8]. The majority of these digitalization efforts pertain to hardware and software solutions that particularly emphasize digitalization on the side of the medical professional and the health care system. Patient access to adequate hardware, the internet, and patient acceptance of digital solutions are mostly assumed to be present in most model projects although it is known that digitalization is largely dependent on factors of age, income, gender, and education [9-13]. The basic requirement for the success of eHealth solutions is not only the “offer” on the side of the health care professionals but also the “demand” on the side of the patient. When implementing a digitalization strategy for a specific question or patient group, it can be assumed that aspects of the degree of the use of digital technical and informal resources of the respective patient cohorts—for example a below-average degree of the use of digital technical and informal resources in the case of an above-average–aged patient cohort—must be paid special attention to [4,12].

The additional benefits of digitalization and the internet are manifold: first, internet use might result in better information concerning breast cancer diagnosis. Li and colleagues [11] showed that patients who used the internet and were satisfied with the internet information concerning their breast cancer diagnosis were significantly more likely to receive breast-conserving therapy and showed significantly improved disease-free survival. Second, the use of online patient-provider communication has increased significantly and might be further developed in order to reach those previously unreached patients [14]. Third, the use of internet-based social community channels might influence patients’ experienced degree of satisfaction with therapy decisions and psychosocial well-being. However, although there is no evidence for a negative impact, the positive effects of online communities have not yet been found to significantly impact patient-reported outcomes, likely because of a large number of influencing factors [15]. One more important secondary result of digitalization may be improved shared decision-making as, for example, communication and contact with other patients is strengthened. Recent studies have evaluated the impact of new technologies on the engagement of patients in shared decision-making and found increased empowerment of patients [16] and the potential for collaborative decision-making [17].

With this paper on patients with breast cancer, we present the first long-term study on the development of the degree of digitalization, including digital device availability and use, stationary and mobile internet access and use, and communication and information seeking regarding the breast cancer diagnosis and treatment of a defined patient group in detail. Using a longitudinal trend study design, we aimed to analyze the development of the most important aspects of digitalization in a well-defined patient cohort. To guide the development of digital study concepts, we aimed to identify subgroups of patients with reduced access to digitalization over the study period spanning 2012 to 2020 who would be excluded from digital patient-physician communication due to their low degree of digitalization.

## Methods

From January 2012 to April 2020 women with a diagnosis of breast cancer were invited to participate in this longitudinal trend study. After a detailed literature search, we developed a questionnaire that included all aspects of the degree of digitalization and the internet use of the patients (Multimedia Appendix 1). In order to make the extent of digitalization more comparable, we summarized the core figures for dealing with digital media into a patient digitalization index (Multimedia Appendix 2).

Statistical analysis was performed using SPSS 25.0 statistical software (IBM Corporation). A *P* value of <.05 was considered significant. Multivariate analysis of age; education; household size; country of origin; and factors of the place of residence including size, rurality, community type, and broadband internet coverage was conducted. This revealed the factors of age, education, and household to be significantly associated with multiple factors of internet ownership and usage. As a consequence, only data concerning these 3 factors are shown.

The study was positively evaluated by the ethics committees of the Universities of Bonn and Cologne and registered in the German Register of Clinical Studies (DRKS00012364).

## Results

A total of 1129 breast cancer patients were interviewed at the breast cancer centers of the University Hospitals of Cologne and Bonn (Center for Integrated Oncology Aachen Bonn Cologne Düsseldorf) in the study period from 2012 to 2020. Of these, 513 patients participated in the study (Multimedia Appendix 3).

### Stationary Device Availability and Use

**Figure 1:**
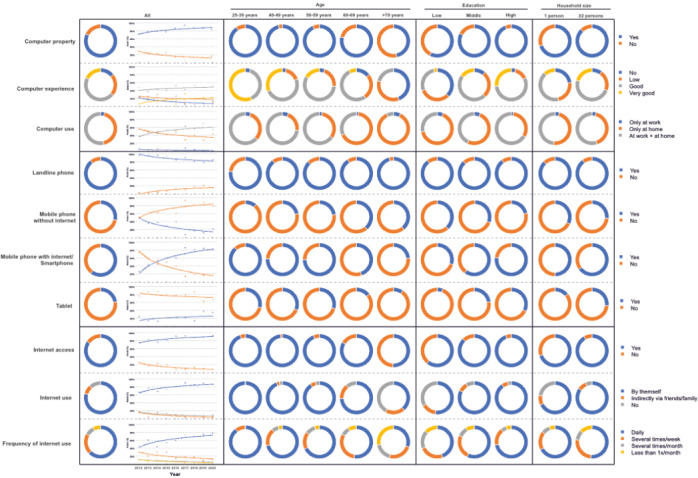
Presence and development of technical and informal resources over the course of the study from 2012 to 2020 in terms of device availability and competence in use, differentiated by age, level of education, and household size. For a higher-resolution version of this figure, see Multimedia Appendix 4.

The basic requirement for access to the internet and the use of eHealth was considered to be the availability of hardware with internet access. Overall, 82.1% (421/513) of patients owned a computer in the study period (Figure 1). The 25 to 59-year-old group showed full computer coverage beginning in 2014 with 94.6% owning computers (279/295). The 60 to 69-year-old group showed a steady increase during the study period, with 83% (10/12) owning a computer in 2020. Only the group of those older than 70 years old showed a smaller increase over the study period, with only half of this patient cohort owning a computer in 2020 (7/13). In addition to age, education was associated with significant differences in computer use: >90% of patients with a high educational background (223/237) had a computer compared to <80% of patients with an educational background lower than high school (198/276; *P*<.001). In addition, a significantly lower portion of patients living alone owned a computer (116/166, 69.9%) compared to patients who lived in a household with at least 2 people (305/347, 87.9%; *P*<.001). We did not observe differences in computer ownership between patients of different origin, place of residence, or broadband coverage.

Furthermore, 64.1% (323/504) of the patients qualified their computer experience as good or very good. Again, patients with a higher education, those under 60 years old, and patients from a household with at least 2 people showed significantly higher computer experience (*P*<.001). Most reported using computers at home (181/422, 42.9%) and/or at work (224/422, 53.1%). Again, younger patients and those with a higher level of education used the computer significantly more both at work and at home (*P*<.001).

### Internet Access and Internet Use

A *conditio sine qua non* for the use of eHealth is access to the internet. Access to the internet at home increased since the beginning of the study and was 84.6% (430/508) at the end of the 9-year study period (Figure 1). Patients <50 years of age showed full coverage of internet access at home since the beginning of the study. A strong increase could be seen in patients aged 50 to 59 years old who had complete access to the internet since 2019. Continuous growth was also evident among those 60 to 69 years old and those older than 70 years, 75% (9/12) and 50% (6/12) of whom were online in 2020, respectively. Patients with different levels of education (primary school education: 71/117, 60.7%; middle school education 135/154, 87.7%; high school education: 224/237, 94.5%) and different household sizes (living alone: 116/166, 69.9%; household size ≥2 people: 314/342, 91.8%) showed significant differences in internet coverage (*P*<.001). Not only the did the availability of the internet at home continuously increase since the beginning of the study, but so did the use of the internet. Moreover, all respondents <40 years old used the internet by themselves since the beginning of the study in 2012, while those 40 to 49 years old and those 50 to 59 years old did so beginning 2016 and 2018, respectively. Continuous growth of internet usage was evident among the 60 to 69-year-old patients and the >70-year-old patients, 63% (5/8) and 50% (6/12) of whom used the internet by 2020, respectively. In addition, significant differences in the use of the internet were observed between patients with different educational backgrounds (primary school education: 59/114, 51.8%; middle school education: 126/153, 82.4%; high school education: 213/236, 90.3%; *P*<.001) and different household sizes (living alone: 111/164, 67.7%; household size ≥2 people: 287/339, 84.7%; *P*<.001). Interestingly, those who were older, had a lower level of education, and who were single used the internet significantly more often indirectly via friends or family, but even more significantly did not use it at all.

### Mobile Internet Access

Although the stationary coverage internet was > 90%, mobile internet access still showed high growth rates. The 25 to 39-year-old group demonstrated full coverage beginning in 2014, while the 40 to 49-year and 50 to 59-year age groups did so beginning in 2016 and 2020, respectively. However, distinct groups still showed a lower access to mobile internet: the 60 to 69-year-old patients (9/12, 75%), the over 70-year-old patients (5/12, 40%), and the patients with little (4/8, 50%) or no education 86% (12/14); meanwhile, in the group of patients with a high school diploma or higher education, this proportion was 100% (13/13). However, a steady increase in mobile internet access was also evident in these patients. As the proportion of patients with mobile or stationary internet access increased, the proportion of patients with either a mobile phone without internet access or landline phone decreased continuously.

### General Information Gathering on Breast Cancer

Digitalization is changing the information resources in cancer and the manner in which this information is accessed. The amount of health-related information on the internet has increased, and the internet has become important for many patients for finding health information. Which sources of information do breast cancer patients generally use to learn about their disease? Which information source is the most important for information? Which source of information influences therapy decision-making (Multimedia Appendix 5)? Over the study period, 74.7% (378/513) of patients saw the treating physicians as the most important information source for their cancer and as the most important information source for therapy decision-making. This did not change over the 9-year study period. For 29.8% (153/513) of patients, the internet was the most important source of information. The proportion of those who use the internet as a source of information increased significantly over the study period in 2012 from 36% (22/61) to 62.5% (40/64). Again, for younger patients with higher education and a partner, the internet was significantly more important as an information resource (*P*<.001). It is important to mention that patients in the year 2020 still considered treating physicians to be the most important source of information on disease and therapy (disease: 45/64, 70%; therapy: 45/60, 75%) as compared to the internet (disease: 30/64, 47%; therapy: 24/60, 40%). Patients without internet access hardly used the internet at all to find information on disease or therapy.

### The Internet as a Source of Information on Breast Cancer

The majority of patients indicated using the internet as a source of information on their disease (Multimedia Appendix 6a and b). In order to determine more precisely how internet use is related to cancer, we asked the patients in detail about their cancer-specific internet use. We found that the internet was used primarily for general information about cancer, for questions about conventional and alternative cancer therapies, for cancer research, and for nutrition in relation to cancer (Multimedia Appendix 6c). In addition, participants indicated using websites of the German Cancer Society (183/286, 62.7%), the German Cancer Aid (174/286, 59.6%), and specialist journals (87/286, 29.8%) to a large extent, while websites of pharmaceutical companies, gynecologists, and patient associations were used much less frequently (each <28/286, <10%). Despite the abundance of information that can be obtained on the internet, 64% (183/286) of patients used the internet only as a source of information in addition to their doctor, and almost no patients (2/286, 0.7%) stated that they did not need any additional information from their doctor besides the internet (Multimedia Appendix 6d). Two-thirds (193/285) of the patients indicated that they had already discussed information about their cancer on the internet with their doctor, 27.1% (79/285) found additional treatment options on the internet, and 15.3% (44/287) stated that this had changed their cancer therapy (Multimedia Appendix 6f). Interestingly, it appears that as soon as a patient uses the internet as a source of information, there exists no differences in search items between patients of different ages, levels of education, or household sizes.

### Reasons Not to Use the Internet to Obtain Information About Cancer

Overall, the proportion of patients that did not use the internet to obtain information decreased continuously beginning from 2012. Those who did not use the internet to obtain information about their illness were significantly older, showed a significantly lower educational background, and significantly more often lived alone. Reasons not to use the internet to find information on their illness mainly included a fear of the information being inaccurate (50/117, 42.7%) or incorrect (62/117, 53%; Multimedia Appendix 6e).

### Association of Internet Access and Therapy Decision

The overwhelming majority of the patients indicated that the decision regarding cancer therapy should either be made by the doctor with knowledge of their preferences (218/483, 45.1%) or on an equal basis within the framework of shared decision-making (154/483, 31.9%; Multimedia Appendix 6g). Only a very small proportion indicated they would like the doctor to decide on cancer therapy alone (25/483, 5.2%). Almost one-fifth of patients indicated that they would like to make this decision themselves, knowing their doctor's recommendation. Overall, these preferences showed no differences across patients of different age groups, different educational levels, different household sizes, or different types of residence. However, patients with internet access and who used the internet wanted to be included significantly more often in the therapy decision-making process (141/218, 64.7%) than did patients without internet access and who did not use the internet (77/218, 35.3%; *P=*.045)

### Communication

Communication over the internet is a basic requirement for many eHealth solutions. In our study, 72.4% (351/485) of patients indicated using the internet for communication (Multimedia Appendix 7a), with the vast majority (340/365, 93.2%) indicating they used it themselves (Multimedia Appendix 7b). Again, those 25 to 49 years old communicated almost completely via the internet, while only 79.7% (118/148) of those 50 to 59 years old, 67.8% (78/115) of those 60 to 69 years old, and 34% (28/82) of those older than 70 years communicated via the internet. Significant differences were observed between patients with high and low levels of education, whereas household size was not associated with differences in communication over the internet.

The vast majority of patients communicated with the oncological outpatient clinics using landline telephones (Multimedia Appendix 7c) although the majority of patients said they would be willing to communicate with their treating physicians by phone (306/415, 73.7%) or email (156/402, 38.8%; Multimedia Appendix 7e and 6f). Additionally, 49.6% (122/246) of patients under 60 years of age compared to 21.8% (34/156) of those over 60 years of age indicated using email as a contact option for oncological outpatient clinics (*P*<.001). Patients with a high level of education used email as a contact option for the oncological outpatient clinics significantly more often (55/226, 24.3%) than did patients with a medium (26/145, 17.9%) or low level of education (7/112, 6.3%; *P*<.001). Access to the internet (access to internet: 88/412, 21.4%; no access to internet:: 0/0, 0%; *P*<.001) and the active use of the internet for information gathering (active use: 75/283, 26.5%; no active use: 12/198, 6.1%; *P*<.001) were significantly associated with the probability of communicating with the oncological outpatient clinics by email.

At the beginning of this study in 2012, few people were able to predict the importance that services such as WhatsApp, Snapchat, or Instagram would have, and it is similarly difficult to predict today the options that will be used in 5 or 10 years. Consequently, openness to new communication options was found to be another important factor in affinity to digitalization of our patients. Importantly, the vast majority of patients (395/430, 91.9%) were willing to use these new communication options (Multimedia Appendix 7g).

### Shopping on the Internet

Even if shopping on the internet does not seem to have a direct connection to the degree of digitalization of breast cancer patients, the diversity of services, in addition to gathering information and communication, including shopping, culture, travel, and delivery and driving services, represents an important aspect of depth in internet offering use (Multimedia Appendix 7d). For instance, 63.6% (300/472) of the participants in the study indicated that they would use the internet themselves for shopping in 2020. Compared to those over 60 years of age (66/189, 34.9%), those under 60 years (234/283, 82.7%) showed significantly higher usage (*P*<.001). Patients with a high level of educational (173/226, 76.5%) also showed a significantly higher usage compared to those with a low level (35/102, 34.3%; *P*<.001) and medium level of communication (92/144, 63.9%; *P*<.001). However, older and less educated patients used the internet for shopping significantly more often indirectly via friends or family. Specifically, 9.2% (26/282) of those under 60 years indicated doing so, while 15.9% (30/185) of those over 60 indicated doing so (*P*=.03). Furthermore, 19.6% (20/102) of patients with a low level of education and 11.1% (16/144) of patients with a medium level of education indicated shopping in this manner, respectively, as compared to 8.9% (20/205) of those with a high educational background (*P*=.02).

### Digitalization Index

The digitalization of breast cancer patients increases every year. This was reflected in the digitalization index of breast cancer patients, which increased from 45 to 55 from 2012 to 2020.

**Figure 2:**
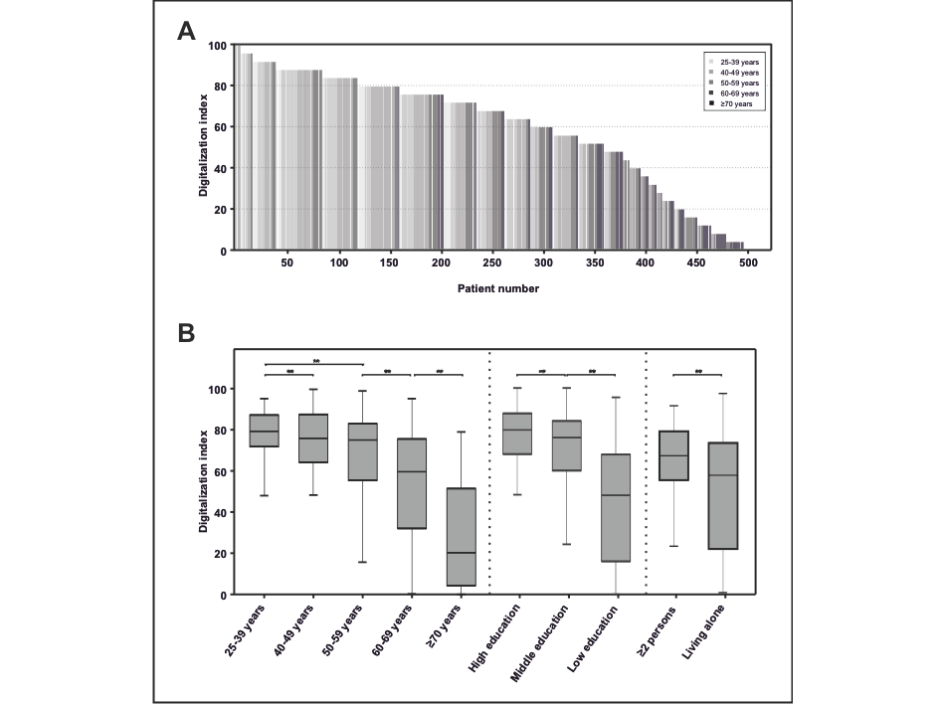
Digitalization index. (A) Waterfall plot of the digitalization index of all participating patients, with the grayscale bar representing age. (B) Digitalization index according to age, level of education, and household size. (**: *P*<.05).

Overall, about 60.4% (310/513) of the patients showed a degree of digitalization of 70 (Figure 2a), and the degree of digitalization decreased significantly with age. In contrast to the high degree of digitalization in the younger age groups, those older than 70 years old, those living alone, and those with less education still showed, despite an increase over the study period, a much lower digitalization index (Figure 2b).

## Discussion

With this longitudinal trend study, we present data on the increase of all aspects of electronic device ownership, internet usage, internet communication, and the influence of the internet on disease information and therapy decision-making in a large cohort of breast cancer patients over a 9-year period.

Digital solutions open up a wide range of possibilities for preventive care, information on disease and therapies, follow-up, and trial support. To succeed, digitalization strategies for distinct clinical questions or patient groups must pay particular attention to specific aspects of the degree of digitalization of the particular patient groups they are designed for.

Our study shows that the availability of electronic devices in breast cancer patients has increased steadily from 2012 to 2020. The same applies to the presence of internet access, internet use, and the availability of mobile devices for internet use. There still are significant differences in terms of both access to and the use of the internet between patients of different ages, educational backgrounds, and household sizes. Patients from a low socioeconomic background, including those older adults living alone and with a low level of education, are significantly less well supplied with internet-enabled devices and with access to the internet. The proportion of patients who do not have internet access and who do not use the internet has decreased steadily since 2012, especially in the group of those older than 70 years old and in the group of those with a low level of educational [9,18].

The internet has become an important source of information for patients [1,9,11,19]. As individual reasons for searching for medical information can vary, for example preparing for a medical consultation, looking up medical information, answering open questions after visiting a doctor, or looking for alternative therapies, understanding how and where patients consume information on cancer on the internet is important to identifying patient needs and offering reputable digital information [9]. Although the proportion of patients that used the internet for information increased in our study, physicians continued to be the most important source of information about disease and therapy throughout the course of the study. In contrast to other countries, the proportion of patients that visit the websites of pharmaceutical companies to search for information is low in Germany [11]. Contrary to our expectations that doctors would be replaced as the most important source of information, it seems that the information seeking on the internet occurs in addition to the physician. The use of information resources others than physicians and the internet seem to decrease over time. Another important result of this study was that there is no difference in the search content among those patients who used the internet as an information resource for understanding their cancer, which suggests that, although the factors age, level of education, and household size are significantly associated with access to the internet and the degree of digitalization, there exist no relevant differences in the type of information being searched for. The increase in knowledge on the side of patients may lead to a more active participation in the decision-making process [19,20]. In our study, the majority of patients had already discussed information from the internet with their doctor, even if this only changed the therapy to a small extent. Access to health-related information can potentially empower patients to be involved in therapy decision-making as compared to the past. In line with other reports on this topic, we observed a difference in therapy decision-making between patients with and without internet use [1-3].

In addition to information acquisition, communication plays a key role in most digitalization solutions. Although the majority of patients in private or professional settings already communicate via the internet, most patients continue to use the phone to contact their doctors. Regardless of the current form of communication with the oncological outpatient clinics, our study showed a steady increase in willingness to communicate with the treating physicians via new communication channels such as email, which has also been shown to be the case in other countries [20].

Some limitations to our study include the relatively small number of patients in the youngest group of people under the age of 40 years that could be recruited for the study in some years. This resulted in a larger SE in this age cohort than in the other age cohorts. In addition, our study recorded the presence of mobile phones and smartphones but did not differentiate between stationary and mobile internet use. However, we do not consider the latter to be a serious drawback since the use of most eHealth applications can be used independently of the device via a browser. As we used a questionnaire to obtain information from the patients, self-reported digital skills could not be assessed objectively and might have been subjectively reported as too high or too low by the patients. In addition, we cannot rule out a potential selection bias in patients that answered the questionnaire compared to those who did not.

Our study offers several insights. Many trials in oncology implement digital solutions, such as electronic patient diaries, video chat functions, or electronic documentation of side effects. Our study identified those patients that would be excluded from such study concepts due to their low degree of digitalization. The planners of trials should keep an eye on the degree of digitalization of their patient population when planning the study in order to ensure that all patient groups have equal access to new trials. Contrary to the fears of some, the internet has not replaced the doctor as an information resource. Our study shows how important it is to provide adequate information on oncological diagnoses on the internet and how wide the scope of information on oncological topics is for the affected patients. However, since a substantial proportion of patients continues to fear incorrect or inaccurate information, the respective physician should guide the patient's search for information, for example by recommending websites with reliable information. The potential benefit of the internet for physician-patient communication in improving clinical care and workflow is likely the largest but also the most underexploited.

We encourage our colleagues to clarify the digitalization status of their patients at the beginning of therapy to optimize digital patient-physician communication.

## Appendix

Multimedia Appendix 1 Questionnaire.

Multimedia Appendix 2 Digitalization index.

Multimedia Appendix 3 Characteristics of 513 patients with breast cancer.

Multimedia Appendix 4 Presence and development of technical and informal resources over the course of the study from 2012 to 2020 in terms of device availability and competence in use, differentiated by age, level of education, and household size.

Multimedia Appendix 5 Most important sources of information and most important sources of information for the treatment of breast cancer.

Multimedia Appendix 6 Use of the internet as source of information.

Multimedia Appendix 7 Use of the internet for communication.
