# Supplementary material for: Digital Technical and Informal Resources of Breast Cancer Patients From 2012 to 2020: Questionnaire-Based Longitudinal Trend Study
Source: JMIR Cancer. 2021 Nov 18;7(4):e20964. doi: 10.2196/20964 (PMC8663592; doi:10.2196/20964)
Supplement: Multimedia Appendix 1 [file cancer_v7i4e20964_app1.docx]

*Multimedia appendix 1: Questionaire*

After a detailed literature search we developed a questionnaire that included all aspects of the degree of digitalization and the internet use of the patients [1-4]. The questionnaire included demographic characteristics such as education, country of origin and relationship status, basic requirements for Internet use such as computer ownership, location and type of computer use, frequency of computer use and sources of information and the importance of different sources of information for the breast cancer diagnosis. The use of the Internet was assessed in four classic areas in which the Internet can be used: information, communication, community and e-commerce and patient characteristics were associated with the data [2, 5].

| **Personal characteristics** |
| --- |
| Education:   - No graduation - High School Graduation - College Graduation - Others: ________________________________   Place of Birth:   - Germany - Middle- and Northern Europe, Northern America - Mediterranean - Eastern Europe - Middle East/ Northern Africa - Asia - Southern America - Africa - Oceania   Marital status   - Married - Single - Widowed - divorced - Living separately - De facto married (living together) - Engaged |

| **Internet Use** |
| --- |
| Do you own a computer?   - yes - no   How do you rate your computer skills?   - no - low - good - very good   Do you use a computer?   - at work - at home - at work + at home   Do you have access to the internet at home?   - yes - no   Do you use the internet?   - yourself - indirectly via relatives/ friends - not     Do you use the internet?   - daily - several times per week - several times per month - ≤1x/ month |

| **General resources of information** |
| --- |
| What types of resources for cancer related information do you use?   - No - Newspaper - Book - Television - Friends/ other patients - General practitioner - Gynecologist - Internet - Others: _____________________________   What type of resources for cancer related information is the most important for you?   - No - Newspaper - Book - Television - Friends/ other patients - General practitioner - Gynecologist - Internet - Others: _____________________________   What type of resources is the most important for you regarding therapy decision making?   - Newspaper - Book - Television - Friends/ other patients - General practitioner - Gynecologist - Internet - Others: _____________________________ |

| Information regarding my disease   - is not important for me - is important for me to gain control over the disease - is important for me to estimate seriousness of the disease - is important for me for „shared decision making“ with my physician |
| --- |

| **Internet as resource of information** |
| --- |
| Do you use the internet as resource for cancer related information?   - yes - no   On a scale from 1 to 7, how interested are you in receiving information from the internet regarding your disease? (1 = no interest to 7 = as much information as possible)  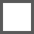 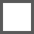 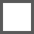 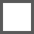 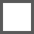 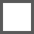 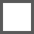  1 2 3 4 5 6 7  If you use the internet as resource of information regarding your disease, what do you search for?   - Information regarding cancer in general - Information regarding cancer-related therapies - Information regarding alternative cancer therapies - Information regarding nutrition and cancer - Information regarding cancer research - Information regarding cancer support community - Information regarding hospitals/ physicians specialized in my disease - Others: _____________________________   If you have Internet access, but you don’t use the Internet as a source of information regarding your disease, that is why?   - I do not find sufficient information regarding my disease in the Internet - I fear inaccurate information regarding my disease in the Internet - I fear wrong information regarding my disease in the Internet - Others: _____________________________ |

| If you currently use the Internet as a source of information regarding your disease, how often do you use the Internet to search for information regarding your disease?   - daily - several times per week - several times per month - <1x/ month   If you currently use the Internet as a source of information regarding your disease, which homepages do you use to search for information regarding your disease?   - Homepage of the gynecologist - Homepage of the hospital - Homepage of the German Cancer Society - Homepage of the German Cancer Aid - Homepage of pharmaceutical companies - Homepage of the scientific journals - Electronic patient associations (for example: Association of Cancer Online Resources etc.) - Other homepages: _____________________________   If you currently use the Internet as a source of information regarding your disease, do you feel adequately informed?   - The Internet offers sufficient information, I do not need additional information from my physician - I feel well informed by the Internet, but I am confused by conflicting information. Here I need assistance from my physician - I do not feel sufficiently informed by the Internet; I need additional information from my physician - I do not feel sufficiently informed by my physician; I need additional information from the Internet - I feel well informed by the physician and do not need additional information from the Internet - I feel well informed by the physician yet search for additional Information in the Internet   If you currently use the Internet as a source of information regarding your disease, have you ever discussed information’s from the Internet regarding your disease, therapy or side effects with your physician ?   - yes - no |
| --- |

| If you currently use the Internet as a source of information regarding your disease, are there information’s from the Internet that have changed your decision regarding the therapy of your disease?   - yes - no   If you currently use the Internet as a source of information regarding your disease, have you found new therapies or trials with novel medications regarding your disease in the internet?   - yes - no   After receiving all the information about their disease and potential treatments, some patients let their physicians decide on their treatment. Other patients want to be involved in the decision-making process. Please state which statement best reflects your attitude towards this:   - My physicians should make the decision on their own based on published studies / guidelines - My physicians should make the decision, but should include my opinion in the decision - My physicians and me should make the decision equally - I would like to make the decisions, but I include the opinion of my doctors in my decision - I would like to make the decisions alone |
| --- |

| **Communication (email, Instant Messaging)/ Community/ e-commerce** |
| --- |
| Do you use the Internet as a medium for communication at the moment (for email, instant messaging, video phone)?   - yes - no   If yes, do you use the Internet as a medium for communication   - yourself - indirectly via friends - indirectly via family   How to you contact our oncologic outpatient clinic?   - no contact - Phone - Email - Instant messaging - Others: _____________________________   Do you use the following Internet based services?  Skype (or other Voice-over-IP services) yes 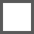 no 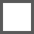 I do not know 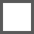  Instant Messaging systems (MSN, Yahoo) yes 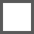 no 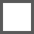 I do not know 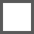  Facetime/ other video phone services yes 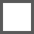 no 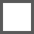 I do not know 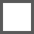  Do you use the Internet for shopping?   - yourself - indirectly via friends - indirectly via family - not - others: _____________________________ |

| **Chemotherapy/ Web 3.0** |
| --- |
| Can you imagine what to expect during chemotherapy?   - yes - no   Would you contact your physician during chemotherapy via phone?   - yes - probably - no   Would you contact your physician during chemotherapy via email?   - yes - probably - no   do you use   - Phone - Mobile phone without internet - Mobile phone with internet / Smartphone - Tablet - Others: _____________________________   Would you use novel technical services with novel ways of communication to contact your physician during therapy?   - yes - probably - no |

**References**

1. Castleton, K., et al., *A survey of Internet utilization among patients with cancer.* Support Care Cancer, 2011. **19**(8): p. 1183-90.

2. Eysenbach, G., *The impact of the Internet on cancer outcomes.* CA Cancer J Clin, 2003. **53**(6): p. 356-71.

3. van de Poll-Franse, L.V. and M.C. van Eenbergen, *Internet use by cancer survivors: current use and future wishes.* Support Care Cancer, 2008. **16**(10): p. 1189-95.

4. van Eenbergen, M., et al., *Changes in internet use and wishes of cancer survivors: A comparison between 2005 and 2017.* Cancer, 2020. **126**(2): p. 408-415.

5. Bundesinstitut für Bau-, S.-u.R., *INKAR - Indikatoren und Karten zur Raum- und Stadtentwicklung*. 2020.
