# Supplementary material for: Digital Technical and Informal Resources of Breast Cancer Patients From 2012 to 2020: Questionnaire-Based Longitudinal Trend Study
Source: JMIR Cancer. 2021 Nov 18;7(4):e20964. doi: 10.2196/20964 (PMC8663592; doi:10.2196/20964)
Supplement: Multimedia Appendix 2 [file cancer_v7i4e20964_app2.docx]

*Multimedia appendix 2: Development of the breast cancer digitalization index*

Digitalization indices are common to combine several factors of digitalization into one number to facilitate comparison of the degree of digitalization of people, societies or countries (e.g. the Euler Hermes Digitalization Index, the D21-Digital-Index in Germany, the IMD World Digital Competitiveness Ranking). 13 questions of our questionnaire that measure the degree of digitalization of patients were combined into a breast cancer patient digitalization index. For this purpose, the various aspects of device conditions, internet comprehensibility and use and the openness of the new, innovative eHealth administration were included: 1. ownership of a computer (0 points = no, 1 point = yes); 2. assessment of one's own computer experience (0 points = no computer experience, 1 point = low computer experience, 2 points = good computer experience, 3 points = very good computer experience); 3. internet access at home (0 points = no, 1 point = yes); 4. usage of the internet (0 points = no, 1 point = indirectly via friends/ family, 2 points = self-usage); 5. frequency of internet usage (0 points = <1x/month, 1 point = several times/ month, 2 points = several times/ week, 3 points = daily); 6. usage of the internet as information source (0 points = no, 1 point = yes); 7. frequency of the internet usage as information source (0 points = <1x/month, 1 point = several times/ month, 2 points = several times/ week, 3 points = daily); 8. usage of the internet for communication (0 points = no, 1 point = yes); 9. usage of the internet for communication (1 point = indirectly via friends/ family, 2 points = self-usage); 10. usage of the internet for shopping (0 points = no, 1 point = indirectly via friends/ family, 2 points = self-usage); 11. willingness to contact the physician via E-mail (0 points = no, 1 point = maybe, 2 points = yes); 12. phone usage (0 points = stationary phone, 1 point = cell phone without internet, 2 points = smartphone); 13. willingness to contact the physician via novel communication channels (0 points = no, 1 point = maybe, 2 points = yes). Each point was multiplied by 4, so in maximum 100 points per patient could be achieved.
