# Supplementary material for: Digital Technical and Informal Resources of Breast Cancer Patients From 2012 to 2020: Questionnaire-Based Longitudinal Trend Study
Source: JMIR Cancer. 2021 Nov 18;7(4):e20964. doi: 10.2196/20964 (PMC8663592; doi:10.2196/20964)
Supplement: Multimedia Appendix 3 [file cancer_v7i4e20964_app3.docx]

*Multimedia appendix 3 - Characteristics of 513 patients with breast cancer*

| Age (spread) |  | 57.3 years (25-85) |
| --- | --- | --- |
|  | Age ≥ 50 years | 72.1% |
|  | Age ≥ 60 years | 42.5% |
|  |  |  |
| Origin | Germany | 89,1% |
|  | Others | 10,9% |
|  |  |  |
| Education | No degree / low educational attainment | 23.4% |
|  | Middle educational attainment | 30.4% |
|  | High school/ College degree | 46.2% |
|  |  |  |
| Household size | Living alone | 32.4% |
|  | Household size ≥ 2 persons | 67.6% |
|  |  |  |
| Type of community | City | 42.3% |
|  | Urban and rural communities | 57.7% |
|  |  |  |
| Type of city/rural area | Large city (>100.000 inhabitants) | 42.3% |
|  | Middle large city | 29.8% |
|  | Small cities and rural communities | 27.9% |
|  |  |  |
| Broadband coverage | Proportion of study participants in localities in which at least 80% of households  - are supplied with ≥16Mbit | 90.4% |
|  | - are supplied with ≥50Mbit | 76.8% |
|  |  |  |
