# Supplementary figures and images for: Digital Technical and Informal Resources of Breast Cancer Patients From 2012 to 2020: Questionnaire-Based Longitudinal Trend Study
Source: JMIR Cancer. 2021 Nov 18;7(4):e20964. doi: 10.2196/20964 (PMC8663592; doi:10.2196/20964)

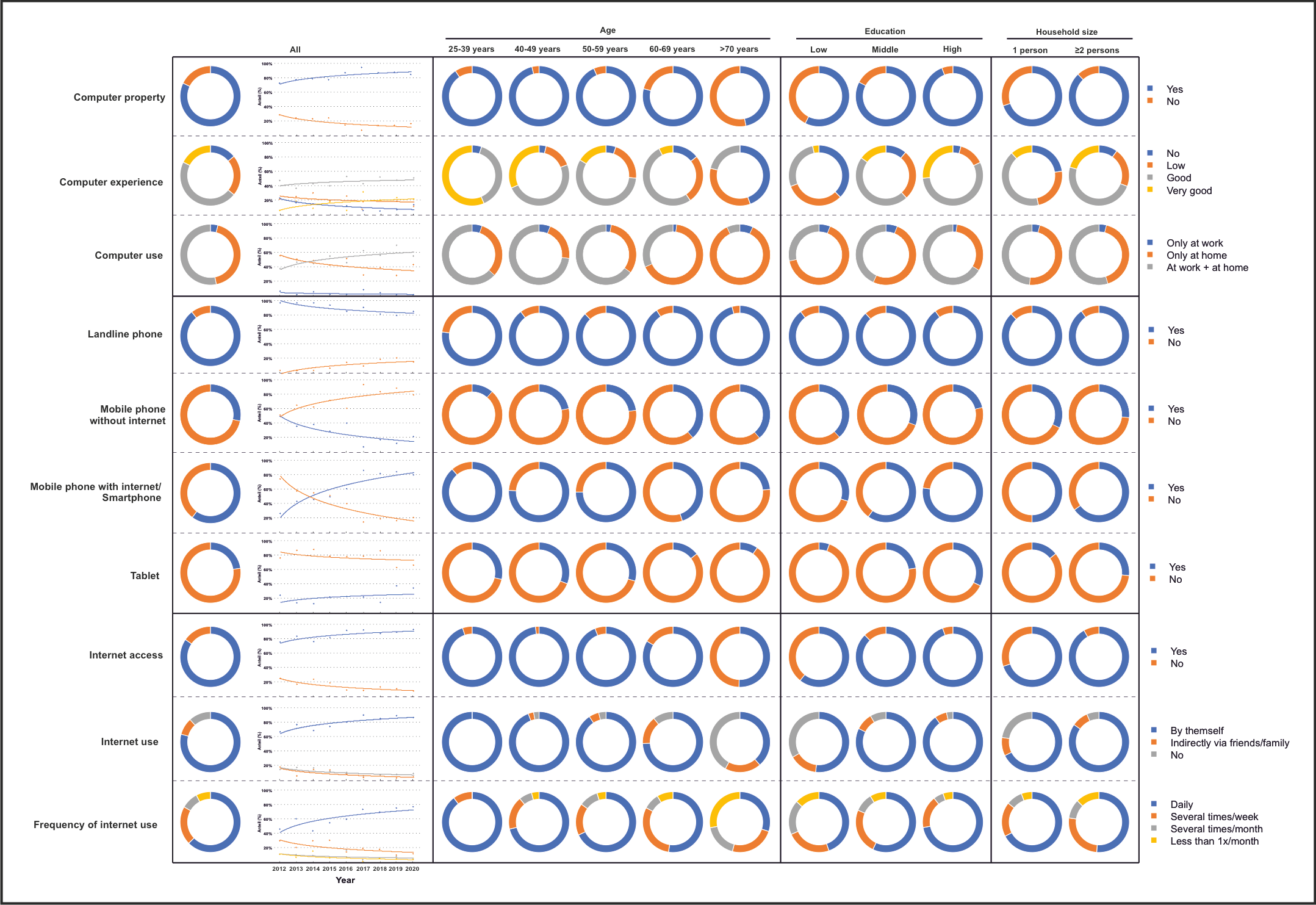

Supplement: Multimedia Appendix 4 [file cancer_v7i4e20964_app4.png]
