# Supplementary material for: Digital Technical and Informal Resources of Breast Cancer Patients From 2012 to 2020: Questionnaire-Based Longitudinal Trend Study
Source: JMIR Cancer. 2021 Nov 18;7(4):e20964. doi: 10.2196/20964 (PMC8663592; doi:10.2196/20964)
Supplement: Multimedia Appendix 5 [file cancer_v7i4e20964_app5.docx]

*Multimedia appendix 4: Most important sources of information on breast cancer*

|  | **Source of information on disease** | **Source of information on treatment** |
| --- | --- | --- |
| Oncologist | 73.7% (378/513) | 76.7% (389/507) |
| Internet | 29.8% (153/513) | 26.4% (134/507) |
| Family doctor | 28.9% (148/513) | 32.2% (163/507) |
| Other patients | 18.9% (97/513) | 16.0% (81/507) |
| Books | 6.4% (33/513) | 4.9% (25/507) |
| Television | 3.5% (18/513) | 2.0% (10/507) |
| Newspaper/ magazines | 6.8% (35/513) | 3.6% (18/507) |
| No resource of information | 2.1% (11/513) | - |
| Others | 17.0% (87/513) | 18.7% (95/507) |
