# Supplementary material for: Digital Technical and Informal Resources of Breast Cancer Patients From 2012 to 2020: Questionnaire-Based Longitudinal Trend Study
Source: JMIR Cancer. 2021 Nov 18;7(4):e20964. doi: 10.2196/20964 (PMC8663592; doi:10.2196/20964)
Supplement: Multimedia Appendix 6 [file cancer_v7i4e20964_app6.docx]

*Multimedia Appendix 5: Use of the internet as source of information.*

| **5a. Use of the internet as source of information** | |  |
| --- | --- | --- |
|  | Yes | 57.4% (292/509) |
|  | |  |
| **5b. Frequency to use the internet as source of information** | |  |
|  | Daily | 6% (53/282) |
|  | Several times per week | 18.8% (53/282) |
|  | Several times per month | 37.6% (106/282) |
|  | <1x/month | 37.6% (106/282) |
|  | |  |
| **5c. Content of the use of the internet as a source of information** | |  |
| **What is searched for in the internet?** | |  |
|  | Information regarding therapies | 66.0% (190/288) |
|  | Information regarding research | 62.2% (179/288) |
|  | General information regarding cancer | 55.6% (160/288) |
|  | Information regarding nutrition and cancer | 50% (144/288) |
|  | Information regarding clinic and specialists | 32.6% (94/288) |
|  | Information regarding alternative therapies | 30.6% (88/288) |
|  | Information regarding support groups | 10.8% (31/288) |
|  | Other informations | 6.3% (18/288) |
| **Which websites are visited?** | |  |
|  | German cancer society | 64% (183/286) |
|  | German cancer aid | 60.8% (174/286) |
|  | Clinic | 42.3% (121/286) |
|  | Oncologic journals | 30.4% (87/286) |
|  | Pharmaceutical companies | 9.4% (27/286) |
|  | Oncologists | 7.3% (21/286) |
|  | Patient support groups | 6.3% (18/286) |
|  | Other websites | 20.4% (57/286) |
|  | |  |
| **5d. Physician versus internet for acquisition of information regarding breast cancer** | | |
|  | There is sufficient information by my physicians, I search for additional information by using the internet | 64% (183/286) |
|  | There is sufficient information by my physicians, I do not need additional information by using the internet | 3.5% (10/286) |
|  | There is insufficient information by my physicians, I need additional information by using the internet | 11.2% (32/286) |
|  | There is insufficient information by using the internet, I need additional information by my physicians | 12.6% (36/286) |
|  | There is sufficient information by using the internet, I need only validation by my physicians | 18.2% (52/286) |
|  | There is sufficient information by using the internet, I do not need additional information by my physician | 0.7% (2/286) |
|  | |  |
| **5e. Reasons not to use the internet to gather information about breast cancer** | |  |
|  | I`m afraid to obtain false information | 53% (62/117) |
|  | I`m afraid to obtain inaccurate information | 50.4% (59/117) |
|  | There is no sufficient information regarding my cancer | 8.5% (10/117) |
|  | Other reasons | 20.5% (24/117) |
|  | |  |
| **5f. The influence of information from the internet for doctor-patient interaction** | |  |
|  | I did discuss findings from the internet with my physician | 67.7% (193/285) |
|  | I found novel information regarding cancer treatment in the internet | 27.7% (79/285) |
|  | I found information in the internet that changed my cancer treatment | 15.3% (44/287) |
|  | |  |
| **5g. Therapy decision** | |  |
|  | My physicians should decide on their own | 5.2% (25/483) |
|  | My physicians should decide upon knowledge of my preferences | 45.1% (218/483) |
|  | Shared-decision making | 31.9% (154/483) |
|  | I decide upon knowledge of my physicians’ recommendations | 17.4% (84/483) |
|  | I decide on my own | 0.4% (2/483) |
|  | |  |
