# Supplementary material for: Digital Technical and Informal Resources of Breast Cancer Patients From 2012 to 2020: Questionnaire-Based Longitudinal Trend Study
Source: JMIR Cancer. 2021 Nov 18;7(4):e20964. doi: 10.2196/20964 (PMC8663592; doi:10.2196/20964)
Supplement: Multimedia Appendix 7 [file cancer_v7i4e20964_app7.docx]

*Multimedia Appendix 6: Use of the internet for communication.*

| **6a. Use of the internet for communication** | |  |
| --- | --- | --- |
|  | Yes | 72.4% (365/504) |
|  | |  |
| **6b. Type of use of the internet for communication** | |  |
|  | Self | 93.2% (340/365) |
|  | Indirect via family | 1.9% (7/365) |
|  | Indirect via friends | 4.9% (18/365) |
|  | |  |
| **6c. Type of communication with the oncological outpatient clinics** | |  |
|  | Telephone | 77.8% (376/483) |
|  | E-Mail | 18.2% (88/483) |
|  | Instant messaging | 0.4% (2/483) |
|  | Others | 2.3% (11/483) |
|  | |  |
| **6d. Type of shopping on the internet** | | |
|  | Self | 63.6% (300/472) |
|  | Indirect via family | 11.9% (56/472) |
|  | Indirect via friends | 1.9% (9/472) |
|  | No | 22.7% (107/472) |
|  | |  |
| **6e. Willingness to communicate with the treating physicians via the telephone** | |  |
|  | Yes | 73.7% (306/415) |
|  | Maybe | 21.2% (88/415) |
|  | No | 5.1% (21/415) |
|  | |  |
| **6f. Willingness to communicate with the treating physicians via E-Mail** | |  |
|  | Yes | 38.8% (156/402) |
|  | Maybe | 24.1% (97/402) |
|  | No | 37.1% (149/402) |
|  | |  |
| **6g. Willingness to communicate with the treating physicians via new forms of communication** | |  |
|  | Yes | 57.7% (246/426) |
|  | Maybe | 31.2% (133/426) |
|  | No | 11% (47/426) |
|  | |  |
